# The Role of Digital Devices in Dentistry: Clinical Trends and Scientific Evidences

**DOI:** 10.3390/jcm9061692

**Published:** 2020-06-02

**Authors:** Gianrico Spagnuolo, Roberto Sorrentino

**Affiliations:** Department of Neuroscience, Reproductive Sciences and Oral Sciences, University of Naples “Federico II”, 80131 Naples, Italy; roberto.sorrentino@unina.it

**Keywords:** digital dentistry, digital tools, technology, computer-aided design/computer-aided manufacturing (CAD/CAM), 3D printing, polyether–ether–ketone (PEEK), digital smile design, guided implant surgery, implant dentistry, restorative dentistry

## Abstract

In recent years, digital technologies have significantlychanged the clinical approach to medicine and dentistry. Innovative operative techniques and restorative materials have paved the way to a significant active boost towards full digital workflows. Particularly, novel dental materials offer undeniable advantages such as optimal mechanical resistance, excellent esthetic and optical properties, and reliable accuracy and precision, widening the clinical scenario and allowing for innovative and less invasive restorative solutions.

Over the past few years, the development of innovative production technologies, performing restorative materials, and novel clinical techniques have pioneered the so-called digital dentistry, broadening treatment options and operative approaches in all branches of dentistry [1,2,3].

Modern technologies have significantly modified our ways of living and working; digital tools have entered our ordinariness powerfully, changing and enhancing the processes of communication, sharing, acquisition, design, and production, with undeniable advantages not only in our working routine [4,5].

As regards the professional perspective and particularly the medical field, such benefits have to be considered in two ways: operator-related and patient-related. Accordingly, digital technologies have significantly contributed to the introduction in several medical branches of innovative conservative techniques, characterized by a meaningful reduction in operative timing and operative invasiveness and by a remarkable improvement in patients’ psychological and physical comfort. Such minimally-invasive approaches have received a strong boost by the innovative solutions offered by digital tools and technologies [6,7,8].

Similarly, the digital workflow came into dentistry in different fields of application, from treatment planning and designing to prototyping steps, from implant surgery procedures to the fabrication of customized prostheses and devices produced by computer-aided design/computer-aided manufacturing (CAD/CAM) additive and subtractive technologies [9,10]. As a consequence, both clinical procedures and laboratory methodologies are moving towards workflows oriented in an increasingly digital way [1].

The introduction of intraoral scanners (IOSs) and advanced fabrication processes such as CAD/CAM technologies and 3D printing has allowed for the implementation of innovative metal-free dental materials, offering the chance to substitute conventional metal frameworks and improving the biomimetic and esthetic outcomes of restorations [1,2]. Moreover, the outstanding mechanical characteristics of these new-generation materials has allowed dentists to reduce the biological sacrifice of bone and dental tissues, reinterpreting the operative procedure in a more conservative way [3].

Technopolymers, hybrid composites, and polycrystalline and high strength ceramics offer undeniable advantages such as excellent mechanical resistance, astounding esthetic and optical properties, reliable precision and accuracy, and at the same time a reduction in chairside and production times [3].

In such a widened scenario, polyether–ether–ketone (PEEK) has gained in popularity and consensus within the scientific and clinical dental communities, thanks to its beneficial characteristics. It is a performing and biocompatible polymer with several applicationsin different areas of medicine and can be used with different purposes and in different configurations [11].

In 2019, Han et al. published an interesting and updated paper on this topic in the Journal of Clinical Medicine entitled “Carbon fiber reinforced PEEK composites based on 3D-printing technology for orthopedic and dental applications”, describing the fabrication of medical devices made up of this material by means of an innovative version of 3D printing technology, namely fused deposition modeling (FDM). In particular, characterized by mechanical testing, the authors produced and compared FDM-printed pure PEEK and carbon fiber-reinforced PEEK composites. Both configurations proved to be highly biocompatible and had no significant surface roughness modifications affecting cell adhesion and cytotoxicity after polishing and sandblasting, but the cell density was higher on samples that did not undergo any surface treatment. Furthermore, the FDM-printed PEEK specimens that were reinforced with carbon fibers showed superior physical properties and a higher mechanical strength than the pure PEEK samples and were proposed as viable material options to fabricate bone grafts and scaffolds [11].

The paper by Han et al. was well designed and interesting, providing abetter understanding of the growing potential of innovative printed technologies and materials in tissue engineering applications that have dramatically changed the clinical scenario of complex maxillofacial and dental treatment planning [11].

Digital tools have significantly changed diagnostic processes (e.g., computed tomography (CT), cone beam computed tomography (CBCT), nuclear magnetic resonance (NMR), ultrasonography, etc.) [12,13] as well as clinical practices (e.g., optical impression, CAD/CAM technologies, stereolithography, 3D printers, etc.) [10]. Particularly in implant surgery, prosthodontics, and restorative dentistry, the introduction of digital planning and previsualization softwares and the use of IOSs have allowed for significative improvements in communication with patients, in the explanation of treatment planning goals, and in patients’ operative and psychological comfort [9,14,15].

Particularly in recent years, digital dental technologies have played a pivotal role in changing the ways of approaching patients and designing innovative and more comprehensive restorative solutions [1,2]. Indeed, digital radiography and data acquisition have offered the possibility of improving diagnostic datasets with the introduction of CBCT [16]. Moreover, 3D fabrication processes (e.g., stereolithography, 3D printing, etc.) and CAD/CAM techniques were implemented in implant dentistry and allowed for the introduction of innovative treatment concepts in dental implant surgery, such as computer-guided implant surgery [10,14]. This approach offered significant simplification and improvements in comparison to traditional surgical techniques, improving the accuracy of implant positioning and making the patients’ comfort and compliance better at the same time [12,13,14].

Furthermore, the introduction of fabrication technologies has permitted the fabrication of prostheses with a full digital workflow [1,2]. The implementation of advanced technologies (e.g., CAD/CAM, laser sintering/melting, 3D printing) has received a synergic boost from the development of novel restorative materials and these improvements have significantly widened the clinical options available for prosthetic treatments in both teeth and implants [3,10].

As a consequence, to date, it is possible to use a full digital workflow from the previsualization of possible surgical and restorative results to the delivery of biocompatible, precise, and highly esthetic restorations, opening up new restorative horizons and working with the so-called “virtual patient” [1,2,17,18].

Operators can take advantage of these improvements related to the digital workflow, performing standardized, easier, and repeatable clinical procedures and in turn improving patients’ comfort and compliance [1,2,19].

Nowadays, innovative technologies and their related digitally-oriented materials have widened the clinical scenario of restorative possibilities; nonetheless, clinicians should always perform updated and informed operative choices based on a comprehensive understanding of the biological, technical, and clinical issues that could affect the outcomes.

Considering the highly dynamic nature of digital dentistry, it is possible to speculate that the technologies available nowadays will be affected by rapid obsolescence and replaced by even more cutting-edge systems and applications. Consequently, constant updates of both the clinical tools and the techniques, as well as the experimental and clinical scientific data, will be necessary to properly understand the further potential development of digital dentistry in the following decades.

## References

[1] Joda T., Zarone F., Ferrari M. (2017). The complete digital workflow in fixed prosthodontics: A systematic review. BMC Oral Health.

[2] Joda T., Ferrari M., Gallucci G.O., Wittneben J.G., Brägger U. (2017). Digital technology in fixed implant prosthodontics. Periodontol. 2000.

[3] Zarone F., Ferrari M., Mangano F.G., Leone R., Sorrentino R. (2016). “Digitally Oriented Materials”: Focus on Lithium Disilicate Ceramics. Int. J. Dent..

[4] Curran V., Matthews L., Fleet L., Simmons K., Gustafson D.L., Wetsch L. (2017). A Review of Digital, Social, and Mobile Technologies in Health Professional Education. J. Contin. Educ. Health Prof..

[5] Bhavnani S.P., Narula J., Sengupta P.P. (2016). Mobile technology and the digitization of healthcare. Eur. Heart J..

[6] Lehne M., Sass J., Essenwanger A., Schepers J., Thun S. (2019). Why digital medicine depends on interoperability. NPJ Digit. Med..

[7] Topol E.J. (2019). A decade of digital medicine innovation. Sci. Transl. Med..

[8] Vandenberghe B. (2018). The digital patient—Imaging science in dentistry. J. Dent..

[9] Cervino G., Fiorillo L., Arzukanyan A.V., Spagnuolo G., Cicciù M. (2019). Dental Restorative Digital Workflow: Digital Smile Design from Aesthetic to Function. Dent. J..

[10] Revilla-León M., Özcan M. (2019). Additive Manufacturing Technologies Used for Processing Polymers: Current Status and Potential Application in Prosthetic Dentistry. J. Prosthodont..

[11] Han X., Yang D., Yang C., Spintzyk S., Scheideler L., Li P., Li D., Geis-Gerstorfer J., Rupp F. (2019). Carbon Fiber Reinforced PEEK Composites Based on 3D-Printing Technology for Orthopedic and Dental Applications. J. Clin. Med..

[12] Colombo M., Mangano C., Mijiritsky E., Krebs M., Hauschild U., Fortin T. (2017). Clinical applications and effectiveness of guided implant surgery: A critical review based on randomized controlled trials. BMC Oral Health.

[13] Zhou W., Liu Z., Song L., Kuo C.L., Shafer D.M. (2018). Clinical Factors Affecting the Accuracy of Guided Implant Surgery-A Systematic Review and Meta-analysis. J. Evid. Based Dent. Pract..

[14] Giordano M., Ausiello P., Martorelli M., Sorrentino R. (2012). Reliability of computer designed surgical guides in six implant rehabilitations with two years follow-up. Dent. Mater..

[15] Moon S.Y., Lee K.R., Kim S.G., Son M.K. (2016). Clinical problems of computer-guided implant surgery. Maxillofac. Plast. Reconstr. Surg..

[16] Pauwels R., Araki K., Siewerdsen J.H., Thongvigitmanee S.S. (2015). Technical aspects of dental CBCT: State of the art. Dentomaxillofac. Radiol..

[17] Bohner L., Gamba D.D., Hanisch M., Marcio B.S., Tortamano Neto P., Laganá D.C., Sesma N. (2019). Accuracy of digital technologies for the scanning of facial, skeletal, and intraoral tissues: A systematic review. J. Prosthet. Dent..

[18] Cervino G., Fiorillo L., Arzukanyan A.V., Spagnuolo G., Campagna P., Cicciù M. (2020). Application of bioengineering devices for stress evaluation in dentistry: The last 10 years FEM parametric analysis of outcomes and current trends. Minerva Stomatol..

[19] Rekow E.D. (2020). Digital dentistry: The new state of the art—Is it disruptive or destructive?. Dent. Mater..

